# Draft Genome Sequence of *Enterococcus raffinosus* Strain CFTRI 2200, Isolated from Infant Fecal Material

**DOI:** 10.1128/genomeA.00932-13

**Published:** 2013-11-07

**Authors:** Kammara Rajagopal

**Affiliations:** Department of Protein Chemistry and Technology (PCT), Central Food Technological Research Institute (CFTRI), a Constituent Establishment of the Council of Scientific and Industrial Research (CSIR), Mysore, India

## Abstract

The complete genome sequence of *Enterococcus raffinosus* strain CFTRI 2200, isolated from the fecal material of a 7-month-old infant, is reported. The complete genome consists of 4.237 Mbp with a G+C content of 39.47% and 4,242 protein-coding genes, 54 tRNAs, and 46 rRNAs.

## GENOME ANNOUNCEMENT

*Enterococcus* bacteria play important roles in food fermentation and are extensively used as probiotic microbes to improve human and animal health (1). These microbes also cause severe nosocomial diseases, urinary tract infections, bacteremia, etc. (2, 3). The present genome sequence may help us to understand *Enterococcus raffinosus* as a potential probiotic microbe.

The whole-genome sequence of the commensal bacterium *E. raffinosus*, which was isolated from fecal material from a healthy Indian (breastfed) infant, is reported here. Sequencing was performed using Illumina Hi Seq 1000 technology (Illumina, Inc., CA). A total of 13,777,040 paired-end reads (insert size 150 bp) of 131-nucleotide lengths were obtained. NGS QC tool kit (v. 2.2.1) was used to filter the data for high quality (HQ) (with 70% cutoff read length and 20% as the quality cutoff score). Vector/adaptor-free reads were used for assembly with Velvet (v. 1.2.07). The final assembly contains 193 contigs, with a total size of 4,207,547 bp and an *N*_50_ contig length of 77.065 kbp. Length of the contigs ranged from 200 bp to 201,862 bp, with a G+C mol% of 39.49%.

Gene prediction was performed by PRODIGAL (Prokaryotic Dynamic Programming Gene finding Algorithm) v. 2.60; tRNAs were predicted using tRNA scan-SE (v. 1.21), and rRNAs were predicted using RNAmmer (v. 1.2). A total of 4,242 genes were predicted, including 54 tRNAs and 7 rRNAs (5S-23S-16S).

The annotation of the genome of *Enterococcus raffinosus* reveals the presence of a Tn*554* element but lacks virulence traits, such as gelatinase (GelE) and serine proteinase (Spr). However, cytotoxins were observed in the genome of *E. raffinosus*, along with phage infection proteins, Mu-like proteins, etc. The genomic region of pathogenicity island 1 (PAI) lacks elements involved in virulence, including cytolysin, compared to V583 or MMH594. This genomic island contains the tetracycline-resistant gene (*tet*), the bile salt hydrolase gene (*bsh*), and lactose metabolic pathway genes (*lacABCDEFG*). However, *E. raffinosus* does not form biofilm but inhibits the formation of biofilm by other strains (*Enterococcus gallinarum*). The *E. raffinosus* genome sequence further emphasizes the presence of the serine threonine kinases Pkn1 and Pkn2, which are similar to the *Mycobacterium tuberculosis* insertion elements IS*6000*, IS*116*, IS*1397*, and IS*1296*, the UvrABC system, and superoxide dismutase (Fe). This genome sequence elucidates the complexity and the genomic differences that distinguish this commensal microbe *E. raffinosus* isolate.

### Nucleotide sequence accession number.

The sequences determined in this study have been deposited with DDBJ/EMBL/GenBank and assigned accession number ATKJ00000000.
